# Hepatitis B Virus Infection in Patients Receiving Allogeneic Hematopoietic Stem Cell Transplantation

**DOI:** 10.3390/jpm11111108

**Published:** 2021-10-28

**Authors:** Yi-Chang Liu, Chi-Mu Hsu, Samuel Yien Hsiao, Hui-Hua Hsiao

**Affiliations:** 1 Division of Hematology and Oncology, Department of Internal Medicine, Kaohsiung Medical University Hospital, Kaohsiung 807, Taiwan; ycliu@cc.kmu.edu.tw (Y.-C.L.); e12013@gmail.com (C.-M.H.); 2 Department of Internal Medicine, Faculty of Medicine, College of Medicine, Kaohsiung Medical University, Kaohsiung 807, Taiwan; 3 Cellular Therapy and Research Center, Kaohsiung Medical University Hospital, Kaohsiung 807, Taiwan; 4 Department of Biology, University of Rutgers-Camden, Camden, NJ 08102, USA; ucdsacnyu@gmail.com; 5 Cancer Center, Kaohsiung Medical University Hospital, Kaohsiung Medical University, Kaohsiung 807, Taiwan

**Keywords:** hepatitis B virus, hematopoietic stem cell transplantation, reactivation, prophylaxis, nucleoside analogues, nucleotide analogues, reverse seroconversion

## Abstract

Considering a steady increase in the number of allogeneic hematopoietic stem cell transplantations (allo-HSCT) worldwide and the significant proportion of the world’s population that has been exposed to hepatitis B virus (HBV) infection, HBV reactivation following allo-HSCT remains an important issue for post-transplant morbidity and mortality. Antiviral prophylaxis can reduce HBV replication, severity of HBV-related hepatitis, and mortality; therefore, identification of patients at risk is crucial. It is recommended that all recipients and donors should be screened for active or prior HBV infection, including HBsAg, antiHBc, and antiHBs. Adoptive immunity transfer from the donor seems to have protective effects against HBV reactivation. Antiviral prophylaxis should be initiated in all HBsAg-positive patients. HBsAg-negative, antiHBc-positive patients remain at risk; therefore, antiviral prophylaxis should be considered if baseline serum HBV DNA is detectable. In HBsAg-negative, antiHBc-positive patients without detectable HBV DNA, close monitoring of viral load with an on-demand therapy is necessary. Entecavir or tenofovir rather than lamivudine are more appropriate for the emergence of lamivudine resistance. The treatment duration remains unclear, with 6- to 12-month therapy after cessation of immunosuppressive therapy commonly recommended. Here we review the updated evidence and recent recommendations regarding HBV reactivation in patients undergoing allo-HSCT for individualized therapy.

## 1. Introduction

Hepatitis B virus (HBV) is a double-stranded DNA virus and is associated with diverse and variable liver diseases in humans ranging from an inactive carrier state to liver cirrhosis and hepatocellular carcinoma. HBV infection is a common disease worldwide; approximately one-third of the world’s population has been exposed to HBV infection, and nearly 350–400 million people are chronically infected by HBV [1]. The prevalence of chronic HBV infection varies widely, ranging from 0.1–20%, with the highest prevalence in sub-Saharan Africa, Southeast Asia, China, and the Pacific islands [1,2]. Infection with HBV is diagnosed by the presence of hepatitis B surface antigen (HBsAg), and in most patients, HBV DNA is also detectable at variable levels. Patients develop antibodies to hepatitis B core antigen (antiHBc) following HBV infection, which persists after clearance of HBsAg and is a marker of current or past HBV infection. Antibodies to hepatitis B surface antigens (antiHBs) are a marker for immunity to HBV and can be present following previous HBV infection or vaccination [3].

The course of HBV infection in humans is determined through the interaction between viral replication and host immune response. The natural history of chronic HBV infection consists of several phases, including immune tolerance, immune clearance, inactive HBV carrier, and HBV reactivation [4,5,6,7]. The immune tolerance phase is characterized by the presence of HBeAg, high HBV DNA level, normal alanine aminotransferase (ALT) level, without evidence of active liver disease. The immune clearance phase is characterized by persistence of hepatitis B e antigen (HBeAg), high or fluctuating HBV DNA level, elevated ALT level, and features of inflammation on liver biopsy. Most patients enter the inactive HBsAg carrier phase, which is characterized by HBeAg seroconversion (absence of HBeAg with presence of antibodies to HBeAg, HBeAb), normal ALT level, and low HBV DNA levels. Then the HBsAg-negative phase may develop, which is characterized by negative HBsAg and positive antiHBc, with or without detectable antiHBs. Patients in this phase have normal ALT values and usually, but not always, undetectable serum HBV DNA. The reactivation phase occurs due to perturbations in the host immune environment, mostly as a result of chemotherapy or immunosuppressive agents, leading to elevation and fluctuation of HBV DNA and ALT level and progressive liver damage on liver biopsy [4,5,6,7].

Allogeneic hematopoietic stem cell transplantation (allo-HSCT) has become the standard curative treatment for many hematological malignancies and non-malignant diseases (e.g., aplastic anemia, hemoglobinopathies, immunodeficiency syndrome). With the improvement of supportive care therapies, conditioning regimens, and donor selection, a steady increase in the number of allo-HSCT treatments worldwide has been observed in the past decades [8,9,10]. Allo-HSCT is an independent risk factor for HBV reactivation in patients with hematological malignancies [11]. Compared with chemotherapy or autologous HSCT, there are several factors that may contribute to an increased risk of HBV reactivation in patients receiving allo-HSCT. These include myeloablative conditioning regimen, graft-versus-host disease (GVHD) prophylaxis strategy (antithymocyte globulin or calcineurin inhibitors), high-dose steroid for GVHD therapy, agents used for chronic GVHD (ruxolitinib, ibrutinib, monoclonal antibodies), and prolonged immune reconstruction following allo-HSCT [12,13]. In places where HBV infection is endemic, the immunosuppressive therapy to HBV-infected patients undergoing allo-HSCT is often associated with a high risk of HBV reactivation, which is sometimes complicated by fulminant hepatic failure leading to mortality. In addition, the adoptive transfer of immunity may happen following allo-HSCT. An HBV-immune/exposed donor has been shown to have a protective role against HBV reactivation following allo-HSCT [14], and clearance of HBsAg has ever been reported after allo-HSCT in patients receiving marrow from antiHBs-positive donors [15].

## 2. HBV Reactivation

Although the definition of HBV reactivation differs between studies, the diagnosis of HBV reactivation is considered when a patient has serological evidence of HBV, in combination with a detectable HBV DNA level if they were previously negative, a rise in HBV DNA of more than 1 or 2 log10 IU/mL compared with baseline HBV DNA level, or a >10-fold increase of HBV DNA level compared with baseline [3,16,17,18,19]. In HBsAg-positive patients, HBV reactivation was defined as a larger than 1 log rise of HBV DNA, compared with baseline level, or detectable HBV DNA in patients whose HBV DNA was previously undetectable. In resolved HBV-infected patients, the HBV reactivation was defined as a positive result of HBsAg in patients who were previously tested HBsAg negative, or a detectable HBV DNA in patients whose HBV DNA were undetectable before. The “reverse seroconversion” is defined in patients with HBsAg-negative, antiHBc-positive who became HBsAg-positive. The rise of HBV DNA level is usually accompanied by an elevation of ALT level and may occur 2–3 weeks earlier before ALT elevation. In 2018, the American Association for the Study of Liver Diseases (AASLD) recommended new criteria for HBV reactivation. For HBsAg-positive patients, one of the followings should be considered: (1) ≥2 log (100-fold) rise in HBV DNA compared to the baseline level, (2) HBV DNA ≥ 3 log (1000) IU/mL in a patient with previously undetectable HBV DNA, (3) HBV-DNA ≥ 4 log (10,000) IU/mL if the baseline level is not available. For HBsAg-negative, antiHBc-positive patients, the criteria that should be used are (1) detection of HBV DNA, (2) reappearance of HBsAg (reverse seroconversion). The diagnostic criteria of HBV reactivation from different recommendations and guidelines are summarized in Table 1. Although reactivation is more common in HBsAg-positive individuals, the prevalence of individuals undergoing allo-HSCT who are antiHBc-positive is much higher than HBsAg-positive individuals. It should be noticed that even though the risk for reactivation is relatively lower in antiHBc-positive individuals, HBV reactivation may be seen more often clinically in this larger at-risk population, particularly in non-endemic settings. It is also worth noting that individual studies may define reactivation as the development of detectable HBV DNA at any level (e.g., >10 IU/mL) in HBsAg-negative, antiHBc-positive individuals. These differences may lead to significant variability in estimates of the cumulative incidence of HBV reactivation among the HBsAg-positive and HBsAg-negative, antiHBc-positive population.

**Table 1:** Diagnostic criteria for HBV reactivation.

Baseline HBV Status	Definition of HBV Reactivation			
	ASCO, 2015 [16]	Paul et al., 2016 [20]	Loomba et al., 2017 [6]	AASLD, 2018 [19]
HBsAg-positive patients				
HBsAg-positive/HBV DNA-positive	HBV DNA ≥ 1 log rise compared with baseline level	HBV DNA ≥ 1 log rise compared with baseline level	HBV DNA ≥ 2 log rise compared with baseline level	HBV DNA ≥ 2 log rise compared with baseline level
HBsAg-positive/HBV DNA-negative	HBV DNA negative to positive	NA	HBV DNA negative to positive	HBV DNA ≥ 1000 IU/mL if previously undetectable HBV DNA ≥ 10,000 IU/mL if baseline not available
Resolved HBV infection patients				
HBsAg-negative/antiHBc-positive/HBV DNA-negative	HBsAg-negative to HBsAg-positive or HBV DNA negative to positive	HBsAg-negative to HBsAg-positive	HBsAg-negative to HBsAg-positive or HBV DNA negative to positive	HBsAg-negative to HBsAg-positive or HBV -DNA negative to positive
HBsAg-negative/antiHBc-positive/HBV DNA-positive	HBsAg-negative to HBsAg-positive	NA	HBsAg-negative to HBsAg-positive	HBsAg-negative to HBsAg-positive

Abbreviations: AASLD, American Association for the Study of Liver Diseases; ASCO, American Society of Clinical Oncology; NA, not available.

When HBV enters hepatocytes, the HBV nucleocapsid is imported into the nucleus, where DNA is converted in the form of the covalently closed circular DNA (cccDNA) [21]. The persistence of cccDNA occurs despite active immune response and serologic clearance of HBV (loss of HBsAg and seroconversion to antiHBs). Therefore, the clinical resolution of acute or chronic HBV infection does not eradicate HBV since cccDNA can persist in infected hepatocytes as a reservoir for HBV reactivation and may become a source of HBV reactivation during immunosuppression [22].

HBV reactivation following cytotoxic chemotherapy or immunosuppressive therapy usually consists of three stages. Initially, HBV replication increases due to immunosuppression caused by chemotherapy or immunosuppressive therapy; at that time, the serum HBV DNA level increases and reflects the degree of viral replication and precedes an elevation of ALT level. In the second stage, the function of the immune system is restored after cessation of chemotherapy or immunosuppressive therapy, and the hepatocytes infected by HBV are cleared. This may lead to an increase of ALT and clinical pictures of acute hepatitis due to hepatocellular injury and may lead to severe liver damage and risk of mortality. In some patients, acute hepatitis may not develop due to the lack of immune reconstitution. In the final recovery phase, clinical hepatitis resolves gradually, and serologic markers return to baseline levels [6,9,23,24,25,26].

The risk of HBV reactivation also depends on the potency of immunosuppressive drugs. Anthracycline derivatives have been associated with a higher risk of HBV reactivation [27,28] and were classified as a high risk in HBsAg-positive patients and a moderate risk in HBsAg-negative, antiHBc-positive patients in the American Gastroenterological Association (AGA) guideline [29]. B-cell depleting agents, such as anti-CD20 monoclonal antibodies, including rituximab and ofatumumab, are considered to carry a high risk of HBV reactivation. A meta-analysis and examination of the FDA safety report revealed that rituximab-based therapy showed an increased risk of HBV reactivation compared with those without rituximab [30]. Rituximab and ofatumumab were classified as a high risk both in HBsAg-positive patients and in HBsAg-negative, antiHBc-positive patients in the AGA guideline [29].

## 3. HBV Serostatus of Donors

The HBV serostatus of the donors also plays a role in HBV reactivation following allo-HSCT. Recipients are at risk of acquiring HBV if allo-HSCT is performed from donors with chronic HBV infection or who have been exposed to HBV. In one early study comparing 18 patients receiving allo-HSCT from HBsAg-positive donors with 18 patients from HBsAg-negative donors at which no prophylaxis was routinely used at that time, eight (44.4%) patients receiving HBsAg-positive donors developed HBV-related hepatitis, including six hepatic failures [31]. A preventive strategy to use lamivudine on donors in the pre-harvest stage was reported among 54 patients receiving allo-HSCT from HBsAg-positive donors, 29 of whom were treated with lamivudine. The HBV-related hepatitis was significantly lower (48% versus 6.9%) in recipients receiving cells from lamivudine-pretreated donors compared with controls [32]. In a large retrospective analysis evaluating the influence of donor/recipient HBV serostatus on allo-HSCT outcomes on 416 recipients from human leukocyte antigen(HLA)-identical related donors, the incidence of treatment-related mortality, survival, GVHD, and hepatic toxicities were similar in 14 HBsAg-positive donors/HBsAg-negative recipients and 28 HBsAg-negative donors/HBsAg-positive recipients receiving antiviral prophylaxis when compared with controls, suggesting that prior exposure to HBV in the donor or recipient is not a contraindication to allo-HSCT [33].

The impact of donor antiHBs status was evaluated in several studies. The adoptive transfer of immunity may happen following allo-HSCT. The clearance of HBsAg has ever been reported after allo-HSCT in patients receiving marrow from antiHBs-positive donors [15]. This is a nearly unique event to HBV and allo-HSCT; besides the very few cases of human immunodeficiency virus that have been cured with allo-HSCT from donors who have specific HLA types, HBV is the only chronic infection that can be cured by transplantation from an immune donor. In a Korean study group, significantly more recipients with resolved HBV infection with antiHBs-negative donors experienced antiHBs loss, and the donor antiHBs showed significant protective effects against the antiHBs loss [34]. In an Italian study group, the risk of HBV reactivation was decreased in the case of an HBV-immune/exposed donor, and the donor immunity was independently associated with a decreased risk of HBV reactivation [14]. In a large retrospective cohort study evaluating 445 allo-HSCT patients with resolved HBV infections, patients with a donor lacking antiHBs was identified as an independent risk factor for HBV reverse seroconversion [35]. These findings imply a mechanism of adaptive immune transfer from the donor and may provide evidence for future donor selection.

## 4. HBV Reactivation Following Allo-HSCT in HBsAg-Positive Patients

Studies from two retrospective cohorts with historical control revealed that the HBV reactivation rate in HBsAg-positive allo-HSCT recipients was 45% without antiviral prophylaxis [36], and 23% and 2% in HBsAg-positive allo-HSCT recipients receiving lamivudine or entecavir, respectively [37]. In one study evaluating the long-term liver complications in HBsAg-positive patients undergoing allo-HSCT, a 19.5% risk of late hepatitis and a 9.8% risk of liver cirrhosis were reported with a median follow-up of 83 months [38]. In addition, HBsAg-positive patients who have a high baseline HBV DNA level were identified to have a high risk of HBV reactivation following HSCT [39]. Factors that have been reported to be associated with the risk of HBV reactivation following allo-HSCT in HBsAg-positive recipients include the serostatus of the donor, HBVDNA level before allo-HSCT, myeloablative conditioning regimen, GVHD prophylaxis strategy (antithymocyte globulin or calcineurin inhibitors), high dose steroid for GVHD therapy, agents used for chronic GVHD, and prolonged immune reconstruction following allo-HSCT [4,39,40]. The use of fludarabine or rituximab as part of conditioning regimen, or T-cell-depleted allograft using anti-thymocyte globulin or alemtuzumab, are associated with an increased risk of HBV reactivation in the early period after allo-HSCT [4,40]. The presence of chronic GVHD, and the treatment of chronic GVHD, including steroids and other immunosuppressive agents and monoclonal antibodies, is also associated with an increased risk of HBV reactivation.

## 5. HBV Reactivation Following Allo-HSCT in HBsAg-Negative, AntiHBc-Positive Patients

It is possible that HBV can potentially have a low replication rate after the resolution of acute HBV infection as evidenced by a detectable serum HBV DNA level in HBsAg-negative, antiHBc-positive patients, which is commonly referred to as occult HBV infection [41]. Therefore, a test for serum HBV DNA levels before allo-HSCT in HBsAg-negative, antiHBc-positive patients is recommended for detection of occult HBV infection [25,42]. In patients with occult HBV infection, the risk of HBV reactivation exists following chemotherapy or immunosuppressive therapy or B-cell depleting agents, such as an anti-CD20 monoclonal antibody, as well as allo-HSCT. In addition, the persistence of cccDNA occurs despite serologic clearance of HBV. Therefore, the clinical resolution of acute or chronic HBV infection does not result in the eradication of HBV since cccDNA can persist in infected hepatocytes as a reservoir for HBV reactivation during immunosuppression [22]. Reverse seroconversion, with the appearance of HBsAg, could be found in HBsAg-negative, antiHBc-positive patients following allo-HSCT, and the risk of HBV reactivation increased cumulatively over time.

In one study, which reported an HBV reactivation rate of 19.7% in HBsAg-negative, antiHBc-positive patients following allo-HSCT, the cumulative probability of HBV reactivation at 1, 2, and 4 years following allo-HSCT was 9%, 21.7%, and 42.9%, respectively, with an association with the presence of chronic GVHD [43]. In another retrospective match-controlled study evaluating the outcomes of HBsAg-negative, antiHBc-positive patients receiving allo-HSCT, the cumulative incidence of HBV reactivation was 11.6% at three years without cases of fulminant hepatic failure. No significant differences were observed in HBsAg-negative, antiHBc-positive patients compared with match-controlled patients in terms of overall survival, relapse rate, non-relapse mortality, and incidence of acute GVHD, suggesting that resolved HBV infection is not associated with poor clinical outcomes [44]. In another study evaluating HBsAg-negative, antiHBc-positive patients receiving allo-HSCT, 12% were found to have reverse seroconversion from 7 to 32 months and were associated with HBeAg-positive hepatitis. Chronic onco-hematological diseases (including chronic myeloid leukemia, chronic lymphocytic leukemia, low-grade lymphoma, and multiple myeloma) and long-lasting immunosuppression after allo-HSCT were identified as risk factors for HBsAg seroconversion [45]. In another study evaluating 137 HBsAg-negative, antiHBc-positive patients receiving allo-HSCT, the competing risk analyses revealed a protective role of an HBV-immune/exposed donor and an increased probability of HBV reactivation associated with the length of treatment with cyclosporine and treatment with rituximab [14]. In another study evaluating 62 HBsAg-negative, antiHBc-positive patients receiving allo-HSCT, the 2-year cumulative HBV DNA detectability rate was 40.8% with a median of 44 weeks. Multivariate analysis showed that age >50 years and chronic GVHD were significantly associated with HBV reactivation [46]. Furthermore, in a large retrospective cohort study evaluating 445 allo-HSCT patients with resolved HBV infection, the cumulative incidence of HBV reserve seroconversion at 3-year and 5-year was 8.7% and 10.5%, respectively, at a median 16 months after allo-HSCT. HBV flares developed in 19% of HBV seroconversion, but none experienced hepatic failure. No impact on non-relapse mortality or survival was identified. Patients with a donor lacking antiHBs and the presence of extensive chronic GVHD were identified as independent risk factors for HBV reverse seroconversion, with a 5-year incidence of 24.2% [35]. A close follow-up protocol with periodic serum HBV DNA level monitoring is needed, especially if such patients could not receive antiviral prophylaxis. The HBV reactivation status in HBsAg-negative, antiHBc-positive patients following allo-HSCT are listed in Table 2.

**Table 2:** HBV reactivation in patients with HBsAg-negative, antiHBc-positive patients receiving allo-HSCT.

Authors	Year	Total Patients	Number of Patients with HBV Reactivation	Onset Time after Allo-HSCT (months), Median (range)	Cumulative Incidence of HBV Reactivation	Risk Factor Analysis	Reference
Hammond et al.	2009	61	12	17.5 (4.4–47)	21.7% at 2 years 42.9% at 4 years	Recipient negative antiHBs Extensive chronic GVHD	[43]
Ramos et al.	2010	73	NR	NR	11.6% at 3 years	NR	[44]
Vigano et al.	2011	50	6	12 (7–32)	13% at 1year 22% at 5 years	Chronic hematological disease Long-lasting immunosuppression	[45]
Park et al.	2011	114	3	27.9 (21–73)	4.8% at 6 years	Donor antiHBs	[34]
Mikulska et al.	2014	137	14	19 (9–77)	9.6% at 3 years 12.2% at 5 years	HBV-immune/exposed donor (less risk) Prolonged cyclosporin use Rituximab treatment	[14]
Seto et al.	2017	62	13	11 (2–25)	17.7% at 1 year 40.8% at 2 years	Age ≥ 50 years Chronic GVHD	[46]
Lee et al.	2019	385	50	19.9 (2.4–75.6)	2.5% at 1 year 57.9% at 7 years	Rituximab treatment	[47]
Bae et al.	2019	69	18	14.7 (2.5–60.9)	11.2% at 1 year 43.0% at 5 years	NR	[48]
Liu et al.	2019	445	21	16 (8–50)	8.7% at 3 years 10.5% at 5 years	Donor lacking antiHBs Extensive chronic GVHD	[35]
Zhang et al.	2020	300	13	21.5 (4.8–65.2)	NR	AntiHBs-negative	[49]

NR, not reported.

## 6. Management and Prophylaxis of HBV Reactivation Following Allo-HSCT

Modulation of immunological environment and control of HBV replication are two key factors in the management of HBV reactivation following allo-HSCT. Although some patients may be asymptomatic at the early stage of HBV reactivation, antiviral therapy should be initiated for all patients who develop HBV reactivation to prevent HBV flare-up, since up to 25–50% of patients may develop severe hepatitis and/or hepatic failure [26]. Prophylactic treatment with antiviral agents can be divided into universal prophylaxis, which is carried out in patients with inactive HBV carriers and/or antiHBc-positive, or targeted prophylaxis, which is carried out in patients with the appearance of HBV DNA and/or HBsAg-positive, or on-demand antiviral therapy, which refers to the initiation of therapy after evidence of HBV reactivation [1,16]. Several drugs have been approved for the treatment of HBV infection worldwide, including interferon (IFN) and nucleoside or nucleotide analogues (NAs). These drugs included two IFN formulations (IFN and pegylated IFN), four nucleosides (lamivudine, telbivudine, entecavir, emtricitabine), and two nucleotides (adefovir, tenofovir) [1].

Lamivudine is one of the NAs that has been studied extensively for its prophylactic role in patients with hematological malignancies receiving chemotherapy or immunosuppressive agents [50,51]. The development of resistance following prolonged lamivudine treatment is clinically important, which is associated with mutations in the YMDD motif of the HBV DNA polymerase gene [42]. In one retrospective study comparing lamivudine and entecavir in 216 HBsAg-positive patients receiving allo-HSCT, the cumulative incidence rates of HBV reactivation at 6, 12, and 24 months following transplantation were 3.0%, 7.0%, and 24.0% in the lamivudine group and 0%, 0%, and 2.0% in the entecavir group, respectively. Mutations leading to drug resistance were detected in 25 patients in the lamivudine group and in only one patient in the entecavir group [37]. In one meta-analysis evaluating the efficacy of antiviral agents against HBV reactivation in allo-HSCT patients, the HBV reactivation rate was 1.9% in patients receiving entecavir and 11.5% in patients receiving lamivudine. Therefore, if prolonged antiviral therapy is expected in patients receiving chemotherapy or B-cell-deleting agents such as an anti-CD20 monoclonal antibody, or immunosuppressive agents for more than 12 months, it is appropriate to avoid lamivudine and to use other NAs with a lower incidence of resistance, such as entecavir or tenofovir [18,19,52]. Considering the high risk of HBV reactivation with prolonged immunosuppressive agents following allo-HSCT, a choice of entecavir or tenofovir is more appropriate rather than lamivudine for its potent inhibition and a high genetic barrier to resistance [18,19,53].

HBsAg-negative, anti-HBc-positive patients had a high rate of HBV reactivation after allo-HSCT, but universal prophylaxis with antiviral agents in HBsAg-negative, antiHBc-positive patients undergoing allo-HSCT remains controversial. In one study investigating the safety and efficacy of entecavir prophylaxis in 57 HBV-infected patients (25 HBsAg-positive, 32 HBsAg-negative/antiHBc-positive) undergoing allo-HSCT, with an intervention to suppress HBV DNA to less than 1000 IU/mL before allo-HSCT and continued antiviral treatment for one year after discontinuation of immunosuppressive therapy, no significant difference was found in HBsAg-positive and HBsAg-negative/antiHBc-positive groups in comparison with the control group in terms of acute or chronic GVHD, drug-induced liver injury, and survival [54]. In one study evaluating 62 HBsAg-negative, antiHBs-positive allo-HSCT patients with frequent HBV DNA monitoring every four weeks up to week 104, the 2-year cumulative HBV DNA detectability rate was 40.8% at a median of 44 weeks. Entecavir at the time of HBV reactivation successfully suppressed HBV DNA to undetectable levels without developing biochemical hepatitis. Patients < 50 years old and without chronic GVHD, compared with the remaining patient cohort, had a significantly lower 2-year cumulative HBV reactivation rate (5.6% versus 65.0%) [46]. The antiviral prophylaxis in selected high-risk patients may be considered if frequent HBV DNA monitoring is not feasible, and further studies are warranted in these groups of patients.

The treatment duration is not well-established in patients undergoing allo-HSCT; however, long-term antiviral treatment may be necessary since patients usually take chronic immunosuppressive medications [12,29,55]. Considering the emergence of lamivudine resistance following prolonged use, entecavir or tenofovir are more appropriate rather than lamivudine as antiviral prophylaxis in the allo-HSCT setting. Furthermore, immune reconstruction-mediated withdrawal hepatitis may occur if antiviral agents were withdrawn together with the cessation of immunosuppressant drugs; the antiviral therapy should be continued for a longer period after the cessation of immunosuppressant drugs [56]. The treatment durations recommended from different guidelines are listed in Table 3.

**Table 3:** Current recommendations for HBsAg-positive and AntiHBc-positive patients receiving allo-HSCT.

	ASCO, 2015 [16]	EASL, 2017 [18]	ESCMID,2017 [57]	AASLD, 2018 [19]	ASCO, 2020 [58]	APASL, 2021 [59]
Screen	HBsAg, antiHBc Classified as high risk	HBsAg, antiHBs, antiHBc Classified as high risk	HBsAg, antiHBs, antiHBc	HBsAg, antiHBc	HBsAg, antiHBs, antiHBc Classified as high risk	HBsAg, antiHBs, antiHBc Classified as high risk
Strategy	Prophylaxis for HBsAg-positive and high-risk HBsAg-negative, Preemptive for low-risk HBsAg-negative	Prophylaxis	Prophylaxis for HBsAg-positive, Prophylaxis for resolved HBV infection	Prophylaxis for HBsAg-positive, Prophylaxis or preemptive for HBsAg-negative/ antiHBc-positive	Start antiviral therapy for HBsAg-positive and HBsAg-negative/ antiHBc-positive	Prophlaxis for HBsAg-positive and HBsAg-negative/ antiHBc-positive
Antiviral drugs	ETV or TDF	ETV, TDF, or TAF	ETV, LAM for HBsAg-positive LAM for resolved HBV infection	ETV, TDF, or TAF	ETV, TDF, or TAF	ETV, TDF, or TAF
Treatment duration	Up to 12 months after cessation of therapy	18 months after stopping immunosuppression	At least 1 year for HBsAg-positive LAM for at least 18 months for resolved HBV infection	Continued at least 12 months after completion of immunosuppressive therapy	Minimum 12 months after anticancer therapy completion	6 months after completion of immunosuppressive therapy

Abbreviations: 3TC, lamivudine; AASLD, American Association for the Study of Liver Diseases; APASL, Asian-Pacific Association for the study of the liver, ASCO, American Society of Clinical Oncology; EASL, European Association for the Study of the Liver; ESCMID, European Society of Clinical Microbiology and Infectious Diseases, ETV, entecavir; HSCT, hematopoietic stem cell transplantation; TAF, tenofovir alafenamide; TDF, tenofovir disoproxil fumarate.

## 7. HBV Vaccination Issues

In European Conference on Infections in Leukemia (ECIL)-7 guideline suggestions for vaccination of allo-HSCT patients, all patients who were HBV-negative at screening or who were vaccinated before HSCT but had a loss of protective immunity six months after HSCT, should undergo HBV vaccination 6–12 months after HSCT with regular antiHBs titer monitoring [60]. In patients with resolved HBV infection, the effectiveness of HBV vaccination to prevent HBV reactivation after allo-HSCT remain unclear. In an early study, antiviral drugs and HBV vaccination after allo-HSCT might prevent reverse seroconversion [61]. In one study enrolling 21 resolved HBV infection allo-HSCT patients, a standard 3-dose regimen of HBV vaccination was arranged after discontinuation of immunosuppressants. Nine tested positive for antiHBs, but none of the 21 patients developed reverse seroconversion [62]. However, in a following prospective study containing 27 resolved HBV infection patients receiving three doses of HBV vaccine 12 months after allo-HSCT [63], 10 tested positive for antiHBs, but the 2-year cumulative incidence of HBV reactivation was 27.3%, which did not show the protective effect in minimizing HBV reactivation. In one study evaluating 118 pediatric and young adult HSCT patients, the antiHBs rate following three doses of HBV vaccination after HSCT was 82% [64]. Another prospective study evaluating 106 patients receiving a lower dose of HBV vaccination (10mg/dose) schedule (three doses one month apart, booster dose 1 year later) six months after allo-HSCT, the proportion of antiHBs titer more than 100 mUI/mL was 64.6% at six months and 56.8% at two years after vaccine initiation [65]. In addition, the vaccination of donors for HBV-positive recipients is suggested with the goal that the adaptive immune response from the vaccinated donor could protect patients from HBV reactivation [66].

## 8. Updated Guideline Recommendations

Currently, there are several practice guidelines recommended by different societies to assist physicians in managing patients receiving allo-HSCT with hepatitis B infection, including the American Society of Clinical Oncology (ASCO) [16,59], American Association for the Study of Liver Disease (AASLD) [19], European Association for the Study Liver (EASL) [18], European Society of Clinical Microbiology and Infectious Diseases (ESCMID) [57], and the Asian-Pacific Association for the study of the liver (APASL) [59]. All guidelines advise screening of HBsAg and antiHBc before allo-HSCT. HBV DNA was suggested in HBsAg-positive patients or in the HBsAg-negative/antiHBc-positive condition in several guidelines. AntiHBs are recommended in several guidelines. In HBsAg-positive patients, all guidelines recommend starting antiviral therapy. In HBsAg-negative, antiHBc-positive patients, some minor discrepancies exist between recommendations regarding the prophylaxis or preemptive strategy. The choice of antiviral agents mainly focuses on entecavir and tenofovir. The treatment duration remains uncertain but is usually more than 12 months. The recommendations are summarized in Table 3.

## 9. Conclusions

HBV reactivation following allo-HSCT is a serious but potentially preventable disease with appropriate treatment; therefore, the identification of patients that are at risk is essential. Antiviral prophylaxis can reduce HBV replication, severity of HBV-related hepatitis, and mortality. It is recommended that all recipients and donors should be screened for active or prior HBV infection, including HBsAg, antiHBc, and antiHBs. Adoptive immunity transfer from the donor seems to have protective effects against HBV-reactivation. The type of allograft, GVHD prophylaxis strategy, long-term immunosuppressive agents, novel therapies such as ibrutinib, ruxolitinib, or monoclonal antibodies may have a potential risk of HBV reactivation and further studies are warranted. HBsAg-positive patients, especially with high HBV DNA levels, have the highest risk of HBV reactivation following allo-HSCT; therefore, antiviral prophylaxis should be initiated in all HBsAg-positive recipients, followed by close monitoring of serum HBV DNA and ALT levels. The benefit of delaying allo-HSCT until a lower HBV DNA level must be weighted with the risk of progression of underlying disease. HBsAg-negative, antiHBc-positive patients remain at risk of HBV reactivation following allo-HSCT; therefore, antiviral prophylaxis should be considered in patients with detectable HBV DNA. For HBsAg-negative, antiHBc-positive patients without detectable HBV DNA, close monitoring of HBV DNA and ALT levels are necessary, and NAs should be initiated if confirmation of HBV reactivation is received. Entecavir or tenofovir rather than lamivudine are more appropriate for antiviral prophylaxis due to the emergence of lamivudine resistance. The treatment duration in the allo-HSCT setting remains unclear, but 6 to 12 months after cessation of immunosuppressive therapy is generally recommended in patients at risk.

## Data Availability

Not applicable.

## References

[1] Orlando R., Foggia M., Maraolo A.E., Mascolo S., Palmiero G., Tambaro O., Tosone G. (2015). Prevention of hepatitis B virus infection: From the past to the future. Eur. J. Clin. Microbiol. Infect. Dis..

[2] Kowdley K.V., Wang C.C., Welch S., Roberts H., Brosgart C.L. (2012). Prevalence of chronic hepatitis B among foreign-born persons living in the United States by country of origin. Hepatology.

[3] Di Bisceglie A.M., Lok A.S., Martin P., Terrault N., Perrillo R.P., Hoofnagle J.H. (2015). Recent US Food and Drug Administration warnings on hepatitis B reactivation with immune-suppressing and anticancer drugs: Just the tip of the iceberg?. Hepatology.

[4] Moses S.E., Lim Z., Zuckerman M.A. (2011). Hepatitis B virus infection: Pathogenesis, reactivation and management in hematopoietic stem cell transplant recipients. Expert Rev. Anti Infect. Ther..

[5] Yapali S., Talaat N., Lok A.S. (2014). Management of hepatitis B: Our practice and how it relates to the guidelines. Clin. Gastroenterol. Hepatol..

[6] Loomba R., Liang T.J. (2017). Hepatitis B Reactivation Associated with Immune Suppressive and Biological Modifier Therapies: Current Concepts, Management Strategies, and Future Directions. Gastroenterology.

[7] Fanning G.C., Zoulim F., Hou J., Bertoletti A. (2019). Therapeutic strategies for hepatitis B virus infection: Towards a cure. Nat. Rev. Drug Discov..

[8] Gratwohl A., Baldomero H., Aljurf M., Pasquini M.C., Bouzas L.F., Yoshimi A., Szer J., Lipton J., Schwendener A., Gratwohl M. (2010). Hematopoietic stem cell transplantation: A global perspective. JAMA.

[9] D’Souza A., Lee S., Zhu X., Pasquini M. (2017). Current Use and Trends in Hematopoietic Cell Transplantation in the United States. Biol. Blood Marrow Transplant..

[10] Copelan E.A., Chojecki A., Lazarus H.M., Avalos B.R. (2019). Allogeneic hematopoietic cell transplantation; the current renaissance. Blood Rev..

[11] Pompili M., Basso M., Hohaus S., Bosco G., Nosotti L., D’Andrea M., Fenu S., Grieco A., Laurenti L., Mirisola C. (2015). Prospective study of hepatitis B virus reactivation in patients with hematological malignancies. Ann. Hepatol..

[12] Gentile G., Antonelli G. (2019). HBV Reactivation in Patients Undergoing Hematopoietic Stem Cell Transplantation: A Narrative Review. Viruses.

[13] Wu Y., Huang H., Luo Y. (2020). Management of Hepatitis B Virus in Allogeneic Hematopoietic Stem Cell Transplantation. Front. Immunol..

[14] Mikulska M., Nicolini L., Signori A., Rivoli G., Del Bono V., Raiola A.M., Di Grazia C., Dominietto A., Varaldo R., Ghiso A. (2014). Hepatitis B reactivation in HBsAg-negative/HBcAb-positive allogeneic haematopoietic stem cell transplant recipients: Risk factors and outcome. Clin. Microbiol. Infect..

[15] Lau G.K., Lok A.S., Liang R.H., Lai C.L., Chiu E.K., Lau Y.L., Lam S.K. (1997). Clearance of hepatitis B surface antigen after bone marrow transplantation: Role of adoptive immunity transfer. Hepatology.

[16] Hwang J.P., Somerfield M.R., Alston-Johnson D.E., Cryer D.R., Feld J.J., Kramer B.S., Sabichi A.L., Wong S.L., Artz A.S. (2015). Hepatitis B Virus Screening for Patients with Cancer Before Therapy: American Society of Clinical Oncology Provisional Clinical Opinion Update. J Clin. Oncol..

[17] Reddy K.R., Beavers K.L., Hammond S.P., Lim J.K., Falck-Ytter Y.T., American Gastroenterological Association Institute (2015). American Gastroenterological Association Institute guideline on the prevention and treatment of hepatitis B virus reactivation during immunosuppressive drug therapy. Gastroenterology.

[18] European Association for the Study of the Liver (2017). Electronic address, e. e. e.; European Association for the Study of the, L. EASL 2017 Clinical Practice Guidelines on the management of hepatitis B virus infection. J. Hepatol..

[19] Terrault N.A., Lok A.S.F., McMahon B.J., Chang K.M., Hwang J.P., Jonas M.M., Brown R.S., Bzowej N.H., Wong J.B. (2018). Update on prevention, diagnosis, and treatment of chronic hepatitis B: AASLD 2018 hepatitis B guidance. Hepatology.

[20] Paul S., Saxena A., Terrin N., Viveiros K., Balk E.M., Wong J.B. (2016). Hepatitis B Virus Reactivation and Prophylaxis During Solid Tumor Chemotherapy: A Systematic Review and Meta-analysis. Ann. Intern. Med..

[21] Hu J., Protzer U., Siddiqui A. (2019). Revisiting Hepatitis B Virus: Challenges of Curative Therapies. J. Virol..

[22] Revill P.A., Chisari F.V., Block J.M., Dandri M., Gehring A.J., Guo H., Hu J., Kramvis A., Lampertico P., Janssen H.L.A. (2019). A global scientific strategy to cure hepatitis B. Lancet Gastroenterol. Hepatol..

[23] Hwang J.P., Lok A.S. (2014). Management of patients with hepatitis B who require immunosuppressive therapy. Nat. Rev. Gastroenterol. Hepatol..

[24] Torres H.A., Davila M. (2012). Reactivation of hepatitis B virus and hepatitis C virus in patients with cancer. Nat. Rev. Clin. Oncol..

[25] Hoofnagle J.H. (2009). Reactivation of hepatitis B. Hepatology.

[26] Lau G.K. (2008). Hepatitis B reactivation after chemotherapy: Two decades of clinical research. Hepatol. Int..

[27] Yeo W., Chan P.K., Hui P., Ho W.M., Lam K.C., Kwan W.H., Zhong S., Johnson P.J. (2003). Hepatitis B virus reactivation in breast cancer patients receiving cytotoxic chemotherapy: A prospective study. J. Med. Virol..

[28] Yun J., Kim K.H., Kang E.S., Gwak G.Y., Choi M.S., Lee J.E., Nam S.J., Yang J.H., Park Y.H., Ahn J.S. (2011). Prophylactic use of lamivudine for hepatitis B exacerbation in post-operative breast cancer patients receiving anthracycline-based adjuvant chemotherapy. Br. J. Cancer.

[29] Choi J., Lim Y.S. (2017). Characteristics, Prevention, and Management of Hepatitis B Virus (HBV) Reactivation in HBV-Infected Patients Who Require Immunosuppressive Therapy. J. Infect. Dis..

[30] Evens A.M., Jovanovic B.D., Su Y.C., Raisch D.W., Ganger D., Belknap S.M., Dai M.S., Chiu B.C., Fintel B., Cheng Y. (2011). Rituximab-associated hepatitis B virus (HBV) reactivation in lymphoproliferative diseases: Meta-analysis and examination of FDA safety reports. Ann. Oncol..

[31] Lau G.K., Lie A.K., Kwong Y.L., Lee C.K., Hou J., Lau Y.L., Lim W.L., Liang R. (2000). A case-controlled study on the use of HBsAg-positive donors for allogeneic hematopoietic cell transplantation. Blood.

[32] Hui C.K., Lie A., Au W.Y., Ma S.Y., Leung Y.H., Zhang H.Y., Sun J., Cheung W.W., Chim C.S., Kwong Y.L. (2005). Effectiveness of prophylactic Anti-HBV therapy in allogeneic hematopoietic stem cell transplantation with HBsAg positive donors. Am. J. Transplant..

[33] Tomblyn M., Chen M., Kukreja M., Aljurf M.D., Al Mohareb F., Bolwell B.J., Cahn J.Y., Carabasi M.H., Gale R.P., Gress R.E. (2012). No increased mortality from donor or recipient hepatitis B- and/or hepatitis C-positive serostatus after related-donor allogeneic hematopoietic cell transplantation. Transpl. Infect. Dis..

[34] Park S., Kim K., Kim D.H., Jang J.H., Kim S.J., Kim W.S., Jung C.W., Koh K.C. (2011). Changes of hepatitis B virus serologic status after allogeneic hematopoietic stem cell transplantation and impact of donor immunity on hepatitis B virus. Biol. Blood Marrow Transplant..

[35] Liu J.H., Liao X.W., Chen C.H., Yao M., Li C.C., Lin C.T., Tsai C.H., Chou W.C., Hou H.A., Huang S.Y. (2019). Adoptive donor immunity protects against resolved hepatitis B virus reactivation after allogeneic haematopoietic stem cell transplantation in the world’s largest retrospective cohort study. Br. J. Haematol..

[36] Lau G.K., He M.L., Fong D.Y., Bartholomeusz A., Au W.Y., Lie A.K., Locarnini S., Liang R. (2002). Preemptive use of lamivudine reduces hepatitis B exacerbation after allogeneic hematopoietic cell transplantation. Hepatology.

[37] Shang J., Wang H., Sun J., Fan Z., Huang F., Zhang Y., Jiang Q., Dai M., Xu N., Lin R. (2016). A comparison of lamivudine vs entecavir for prophylaxis of hepatitis B virus reactivation in allogeneic hematopoietic stem cell transplantation recipients: A single-institutional experience. Bone Marrow Transplant..

[38] Hui C.K., Lie A., Au W.Y., Leung Y.H., Ma S.Y., Cheung W.W., Zhang H.Y., Chim C.S., Kwong Y.L., Liang R. (2005). A long-term follow-up study on hepatitis B surface antigen-positive patients undergoing allogeneic hematopoietic stem cell transplantation. Blood.

[39] Lau G.K., Leung Y.H., Fong D.Y., Au W.Y., Kwong Y.L., Lie A., Hou J.L., Wen Y.M., Nanj A., Liang R. (2002). High hepatitis B virus (HBV) DNA viral load as the most important risk factor for HBV reactivation in patients positive for HBV surface antigen undergoing autologous hematopoietic cell transplantation. Blood.

[40] Moses S.E., Lim Z.Y., Sudhanva M., Devereux S., Ho A.Y., Pagliuca A., Zuckerman M., Mufti G.J. (2006). Lamivudine prophylaxis and treatment of hepatitis B Virus-exposed recipients receiving reduced intensity conditioning hematopoietic stem cell transplants with alemtuzumab. J. Med. Virol..

[41] Sugauchi F., Tanaka Y., Kusumoto S., Matsuura K., Sugiyama M., Kurbanov F., Ueda R., Mizokami M. (2011). Virological and clinical characteristics on reactivation of occult hepatitis B in patients with hematological malignancy. J. Med. Virol..

[42] Lalazar G., Rund D., Shouval D. (2007). Screening, prevention and treatment of viral hepatitis B reactivation in patients with haematological malignancies. Br. J. Haematol..

[43] Hammond S.P., Borchelt A.M., Ukomadu C., Ho V.T., Baden L.R., Marty F.M. (2009). Hepatitis B virus reactivation following allogeneic hematopoietic stem cell transplantation. Biol. Blood Marrow Transplant..

[44] Ramos C.A., Saliba R.M., de Padua Silva L., Khorshid O., Shpall E.J., Giralt S., Patah P.A., Hosing C.M., Popat U.R., Rondon G. (2010). Resolved hepatitis B virus infection is not associated with worse outcome after allogeneic hematopoietic stem cell transplantation. Biol. Blood Marrow Transplant..

[45] Vigano M., Vener C., Lampertico P., Annaloro C., Pichoud C., Zoulim F., Facchetti F., Poli F., Scalamogna M., Deliliers G.L. (2011). Risk of hepatitis B surface antigen seroreversion after allogeneic hematopoietic SCT. Bone Marrow Transplant..

[46] Seto W.K., Chan T.S., Hwang Y.Y., Wong D.K., Fung J., Liu K.S., Gill H., Lam Y.F., Lau E.H.Y., Cheung K.S. (2017). Hepatitis B reactivation in occult viral carriers undergoing hematopoietic stem cell transplantation: A prospective study. Hepatology.

[47] Lee H.L., Jang J.W., Han J.W., Lee S.W., Bae S.H., Choi J.Y., Han N.I., Yoon S.K., Kim H.J., Lee S. (2019). Early Hepatitis B Surface Antigen Seroclearance Following Antiviral Treatment in Patients with Reactivation of Resolved Hepatitis B. Dig. Dis. Sci..

[48] Bae S.K., Gushima T., Saito N., Yamanaka I., Matsuo Y., Yoshida S., Kawano I., Henzan H., Shimoda S., Eto T. (2019). HBV reactivation after hematopoietic stem cell transplantation and rituximab-containing chemotherapy: A 12-year experience at a single center. Bone Marrow Transplant..

[49] Zhang A., Wu Y., Tan Y., Shi J., Zhao Y., Hu Y., Yu J., Zheng W., Lai X., Zhang M. (2020). Determining Whether Prophylactic Antiviral Treatment Is Necessary in HBsAg-Negative/HBcAb-Positive Patients Receiving Allogeneic Hematopoietic Stem Cell Transplantation. Biol. Blood Marrow Transplant..

[50] Manzano-Alonso M.L., Castellano-Tortajada G. (2011). Reactivation of hepatitis B virus infection after cytotoxic chemotherapy or immunosuppressive therapy. World J. Gastroenterol..

[51] Liang R. (2009). How I treat and monitor viral hepatitis B infection in patients receiving intensive immunosuppressive therapies or undergoing hematopoietic stem cell transplantation. Blood.

[52] Lok A.S., McMahon B.J. (2007). Chronic hepatitis B. Hepatology.

[53] Santantonio T.A., Fasano M. (2014). Chronic hepatitis B: Advances in treatment. World J. Hepatol..

[54] Liao Y.P., Jiang J.L., Zou W.Y., Xu D.R., Li J. (2015). Prophylactic antiviral therapy in allogeneic hematopoietic stem cell transplantation in hepatitis B virus patients. World J. Gastroenterol..

[55] Gentile G., Andreoni M., Antonelli G., Sarmati L. (2017). Screening, monitoring, prevention, prophylaxis and therapy for hepatitis B virus reactivation in patients with haematologic malignancies and patients who underwent haematologic stem cell transplantation: A systematic review. Clin. Microbiol. Infect..

[56] Hui C.K., Cheung W.W., Au W.Y., Lie A.K., Zhang H.Y., Yueng Y.H., Wong B.C., Leung N., Kwong Y.L., Liang R. (2005). Hepatitis B reactivation after withdrawal of pre-emptive lamivudine in patients with haematological malignancy on completion of cytotoxic chemotherapy. Gut.

[57] Sarmati L., Andreoni M., Antonelli G., Arcese W., Bruno R., Coppola N., Gaeta G.B., Galli M., Girmenia C., Mikulska M. (2017). Recommendations for screening, monitoring, prevention, prophylaxis and therapy of hepatitis B virus reactivation in patients with haematologic malignancies and patients who underwent haematologic stem cell transplantation-a position paper. Clin. Microbiol. Infect..

[58] Hwang J.P., Feld J.J., Hammond S.P., Wang S.H., Alston-Johnson D.E., Cryer D.R., Hershman D.L., Loehrer A.P., Sabichi A.L., Symington B.E. (2020). Hepatitis B Virus Screening and Management for Patients with Cancer Prior to Therapy: ASCO Provisional Clinical Opinion Update. J. Clin. Oncol..

[59] Lau G., Yu M.L., Wong G., Thompson A., Ghazinian H., Hou J.L., Piratvisuth T., Jia J.D., Mizokami M., Cheng G. (2021). APASL clinical practice guideline on hepatitis B reactivation related to the use of immunosuppressive therapy. Hepatol. Int..

[60] Cordonnier C., Einarsdottir S., Cesaro S., Di Blasi R., Mikulska M., Rieger C., de Lavallade H., Gallo G., Lehrnbecher T., Engelhard D. (2019). Vaccination of haemopoietic stem cell transplant recipients: Guidelines of the 2017 European Conference on Infections in Leukaemia (ECIL 7). Lancet Infect. Dis..

[61] Le Q., Shenoy A., Koklanaris E., Childs R., John Barrett A., Savani B.N. (2009). Lamivudine prophylaxis and hepatitis B vaccination for prevention of hepatitis B virus reverse seroconversion in long-term survivors after allogeneic stem cell transplantation. Biol. Blood Marrow Transplant..

[62] Takahata M., Hashino S., Onozawa M., Shigematsu A., Sugita J., Fujimoto K., Endo T., Kondo T., Tanaka J., Imamura M. (2014). Hepatitis B virus (HBV) reverse seroconversion (RS) can be prevented even in non-responders to hepatitis B vaccine after allogeneic stem cell transplantation: Long-term analysis of intervention in RS with vaccine for patients with previous HBV infection. Transpl. Infect. Dis..

[63] Nishikawa K., Kimura K., Kanda Y., Sugiyama M., Kakihana K., Doki N., Ohashi K., Bae S.K., Takahashi K., Ishihara Y. (2020). A prospective trial of vaccine to prevent hepatitis B virus reactivation after hematopoietic stem cell transplantation. Bone Marrow Transplant..

[64] Chaichotjinda K., Anurathapan U., Boonsathorn S., Chaisavaneeyakorn S., Treepongkaruna S., Techasaensiri C., Apiwattanakul N. (2020). Immune responses to hepatitis B vaccination after hematopoietic stem cell transplantation in pediatric and young adult patients. Clin Transplant..

[65] Conrad A., Perry M., Langlois M.E., Labussiere-Wallet H., Barraco F., Ducastelle-Lepretre S., Larcher M.V., Balsat M., Boccard M., Chidiac C. (2020). Efficacy and Safety of Revaccination against Tetanus, Diphtheria, Haemophilus influenzae Type b and Hepatitis B Virus in a Prospective Cohort of Adult Recipients of Allogeneic Hematopoietic Stem Cell Transplantation. Biol. Blood Marrow Transplant..

[66] Lindemann M., Koldehoff M., Fiedler M., Schumann A., Ottinger H.D., Heinemann F.M., Roggendorf M., Horn P.A., Beelen D.W. (2016). Control of hepatitis B virus infection in hematopoietic stem cell recipients after receiving grafts from vaccinated donors. Bone Marrow Transplant..

